# Relationships Among eHealth Literacy, Physical Literacy, and Physical Activity in Chinese University Students: Cross-Sectional Study

**DOI:** 10.2196/56386

**Published:** 2024-11-04

**Authors:** Shan Jiang, Johan Y Y Ng, Siu Ming Choi, Amy S Ha

**Affiliations:** 1 Department of Sport Science and Physical Education The Chinese University of Hong Kong Hong Kong China (Hong Kong); 2 Faculty of Education University of Macau Macau China

**Keywords:** eHealth literacy, physical literacy, physical activity, university students, health behavior

## Abstract

**Background:**

eHealth literacy is critical for evaluating abilities in locating, accessing, and applying digital health information to enhance one’s understanding, skills, and attitudes toward a healthy lifestyle. Prior research indicates that enhancing eHealth literacy can improve health behaviors such as physical activity (PA). Physical literacy (PL) refers to the ability to develop sustainable PA habits, taking into account various aspects of an individual. Notably, university students have shown a decline in PA and possess low PL levels. However, the connection between eHealth literacy and PL in this demographic has not been extensively studied, and it remains uncertain whether PA acts as a mediator between eHealth literacy and PL.

**Objective:**

This study examines the extent to which PA mediates the link between eHealth literacy and PL in Chinese university students and explores gender differences in these variables.

**Methods:**

In February 2022, a cross-sectional survey was administered to 1210 students across 3 universities in China. The instruments used were the Perceived PL Instrument, the International Physical Activity Questionnaire, and the Chinese version of the eHealth Literacy Scale. Correlations between eHealth literacy, PA, and PL were analyzed using Pearson product-moment correlation and multiple linear regression, while mediation models helped elucidate the interactions among the 3 variables.

**Results:**

The response rate for the study was 92.9% (1124/1210). In the mediation analysis, eHealth literacy showed a significant direct effect on PL, with a coefficient of 0.78 (β .75, SE 0.02; *P<.*001). Moderate to vigorous physical activity (MVPA) accounted for 2.16% of the total effect, suggesting that MVPA partially mediates the relationship between eHealth literacy and PL. Additionally, male students outperformed female students in terms of MVPA (*t*_636_=4.94; *P<.*001) and PL (*t*_636_=3.18; *P<.*001), but no significant differences were found in eHealth literacy (*t*_636_=1.23; *P*=.22).

**Conclusions:**

The findings indicate that MVPA serves as a mediator in the link between eHealth literacy and PL among university students. Students with low eHealth literacy or limited PA are less likely to be physically literate. Thus, eHealth literacy plays a crucial role in enhancing PL and PA, especially when interventions targeting PL are implemented. Our results also suggest a need for targeted health education interventions aimed at improving MVPA and PL among female students, while also recognizing that eHealth literacy is comparable across genders at universities.

## Introduction

### Physical Activity and Health

In recent times, health challenges have escalated into a global issue. Physical activity (PA) is consistently recognized for its benefits to individual health and as a preventative measure for prevalent noncommunicable diseases. The World Health Organization (WHO) advocates for adults to partake in a minimum of 150 minutes per week of moderate physical activity (MPA) [1,2]. Nonetheless, it is reported that about 24% of young adults have not met these PA guidelines and are in urgent need to address the health risks associated with inadequate PA [3]. A meta-analysis indicates that physical inactivity affects 40% to 50% of university students [4]. Particularly in mainland China, a significant decline in PA has been observed among university students. Studies have identified that the prevalence of engaging in less than 1 hour of PA is highest among Chinese men aged 18 years and women aged 21 years, with rates of 82.5% and 89.8%, respectively [5]. Furthermore, the proliferation of the internet and increased use of electronic devices, such as video games and smartphones, have significantly decreased PA time and frequency [6-8]. Consequently, enhancing PA levels among university students, effectively using web-based resources, and boosting their health awareness are imperative in promoting a culture of wellness, reducing sedentary behaviors, and fostering lifelong habits of healthy living. In addition, moderate to vigorous physical activity (MVPA) plays a crucial role in the overall health and well-being of young adults. Research consistently highlights the significance of MVPA in this demographic due to its profound impact on cardiovascular health [9,10], cognitive function [11,12], and mental health [13]. Engaging in MVPA not only helps to maintain a healthy weight and reduce the risk of chronic diseases but also enhances overall physical fitness and endurance [9]. Moreover, MVPA has been linked to improved emotional regulation, stress reduction, and better sleep patterns, all of which are vital aspects of mental health in young adults and their late life [13,14].

### eHealth Literacy

Electronic resources have become integral in disseminating health knowledge and play a pivotal role in public health [15]. Coined by Norman and Skinner [16], “eHealth literacy” refers to the capacity of individuals to search, comprehend, and assess basic health information from the internet, and to apply such knowledge in solving health-related issues [17]. University students are key disseminators of health knowledge and practitioners of health behaviors [18]. They are also primary consumers of digital information, making their understanding of eHealth crucial for societal impact. Numerous studies indicate that eHealth literacy among university students is shaped by factors such as educational environment, gender, and familial background [19,20].

Concerning differences in educational background, medical students typically exhibit higher eHealth literacy and more accessible electronic health information than nonmedical students [18]. Recent findings suggest that men display higher eHealth literacy compared to women [21,22]. Moreover, eHealth literacy has been associated with better health status and healthier behaviors including regular consumption of healthy foods and consistent PA [23-26]. Those with enhanced eHealth literacy are more inclined to participate regularly in PA [27]. However, the exploration of eHealth literacy in China remains nascent. Few studies have investigated the link between eHealth literacy and PA among university students [22,28] including the variations in eHealth literacy among students in health-related fields such as medicine and sports science.

### Physical Literacy

Physical literacy (PL) encompasses the skills and attributes demonstrated by individuals through PA and movement throughout their life span [29]. It is both a process and outcome pursued through an amalgamation of physical, emotional, social, and cognitive interactions [30,31]. These interconnected domains foster the comprehensive development of PL, aiding individuals of all ages to lead active, healthy, and fulfilling lives. Additionally, the WHO has emphasized the integration of PL with health promotion and the enhancement of PA in national public health strategies [32]. Previous research underscores that PL is fundamental to PA, and individuals with low PL are less likely to engage in substantial PA [33-35]. Moreover, advancements in internet technologies, such as active video games and social media, have positively influenced students’ PL [36-38]. Thus, it is crucial to improve PL among university students and focus on the interrelation between eHealth literacy and PL. Notably, the link between eHealth literacy and PL has yet to be fully explored.

### The Mediating Role of PA in the Relationship Between eHealth Literacy and PL

Delving into the complex relationships among eHealth literacy, PL, and PA is essential. PA acts as a crucial behavioral mediator through which eHealth literacy influences PL. Elevated eHealth literacy enables individuals to access a variety of digital resources, apps, and wearable technologies that encourage PA [39] participation. Web-based platforms also provide instructional content that educates users on proper exercise techniques, the benefits of PA, and methods to integrate movement into daily routines. This information aids individuals in acquiring the necessary skills and knowledge of diverse physical activities, thereby boosting their motivation and self-efficacy for PA engagement, which enhances overall PL [40].

Participation in PA yields cognitive advantages, such as improved memory, attention, and executive functions, enhancing the ability of individuals to understand and apply health-related information from eHealth platforms [41,42]. This synergy between eHealth literacy and PL is further strengthened by the social support found in PA activities, characterized by community interaction and teamwork. Such social engagement is especially beneficial for university students, promoting consistent PA participation. With higher eHealth literacy, individuals gain better access to eHealth resources including social functionalities that enable connection with peers, group participation, and involvement in community challenges [43]. This social integration fosters a sense of community and accountability, encouraging individuals to remain active and enhance their PL through collective support and motivation.

### Gender Differences in PA, eHealth Literacy, and PL

Exploring the disparities in health behaviors and literacy across genders is crucial [15,44]. Prior research indicates that men typically engage in higher levels of PA than women among adolescents and young adults, influenced by societal norms, resource availability, and personal inclinations [45]. Additionally, variations in eHealth literacy have been observed between genders, with some studies suggesting that women may exhibit higher eHealth literacy than men [22,39,46]. Concerning PL, prior studies have explored potential gender-based disparities in its development, pinpointing unique strengths and challenges for men and women [47-49]. These findings underscore the importance of tailored educational interventions to bolster PL within distinct gender cohorts [40,50]. Understanding these gender differences is pivotal for crafting targeted strategies to promote overall health and wellness among both men and women.

Thus, the primary objective of this study is to examine the mediating role of PA between eHealth literacy and PL among Chinese university students. We also aimed to assess how individual factors influence these variables such as gender. MVPA, as a key component of PA, plays a crucial role in promoting overall health development [1,3]. Thus, we hypothesized that eHealth literacy is positively associated with university students’ MVPA (H1); MVPA will mediate the relationship between eHealth literacy and PL (H2); and gender differences may exist in eHealth literacy, MVPA, and PL among Chinese university students (H3).

## Methods

### Recruitment

A cross-sectional survey was conducted using Wenjuanxing, a popular Chinese web-based questionnaire platform, from January to February 2022. Initially, a pilot test was undertaken with 89 sophomore students from Beijing Normal University to assess the readability and item wording of the questionnaire. Feedback was gathered, and after a preliminary analysis, the questionnaire was revised accordingly.

Participants were enlisted through a snowball sampling approach. A recruitment notice was circulated via WeChat, a widely used social media platform in mainland China. Six university students, who were active members of their university student associations, were purposively chosen from 3 prominent Chinese universities—Beijing Normal University, Beijing Jiaotong University, and Beijing University of Chinese Medicine—following a rigorous selection process. These students were selected due to their regular involvement in university activities, which provided them with the capacity to reach various academic groups within their institutions. Following their anonymous participation in the survey, these students invited their peers and other university students to partake in the study through WeChat in Chinese. Importantly, there was no overlap of participants between the pilot and main studies to minimize bias.

Using parameters such as a total student body of 40,000 at each university, a 5% margin of error, a 95% CI, and a 50% response rate assumption, the necessary sample size was calculated using Raosoft [51], resulting in an optimal sample size of 381 participants.

### Participants

If participants agreed to participate, they could click the “Start the Survey” button to proceed with the questionnaire; those who chose not to participate could simply click the “Close” button. Eligible participants included Chinese-speaking, full-time undergraduates or postgraduates aged 18-28 years at the specified universities. Although previous studies have indicated varying age ranges for Chinese university students [28,52-55], we have reviewed the current situation in Chinese universities and found that the majority of undergraduate and postgraduate students complete their studies between the ages of 18 and 28, which is the typical university age. This age range is crucial for understanding the dynamics of eHealth literacy, PL, and PA, as these factors are often influenced by the educational and developmental milestones typical for this demographic. The exclusion criteria were (1) individuals with missing or incomplete survey data, (2) respondents who provided inconsistent or implausible answers, (3) those who did not comply with the survey completion guidelines, and (4) individuals with medical conditions or physical constraints that might affect participation.

### Ethical Considerations

This study received approval from the Survey and Behavioral Research Ethics Committee of The Chinese University of Hong Kong (EDU2022-069). Prospective participants accessed the survey via a link and were initially presented with an informed consent form. The purpose of both the survey and the informed consent form was clearly stated on the first page of the questionnaire. We ensured that participants’ information would be kept confidential and anonymous. Participation was voluntary, and no financial incentives were offered.

### Instruments and Measurements

#### eHealth Literacy

The main measure of this study was the Chinese version of the eHealth Literacy Scale (eHEALS). It comprises 8 items, with responses ranging from 1 (strongly disagree) to 5 (strongly agree), totaling a possible 40 points [56,57]. The scale was adapted following WHO guidelines, and its reliability was confirmed (Cronbach α=0.85) [56].

#### PL Level

Perceived PL among Chinese university students was evaluated using the Perceived Physical Literacy Instrument (simplified Chinese version) [35]. This instrument includes 8 items distributed across 3 dimensions: confidence and physical competence (eg, “I am physically fit, in accordance with my age”), motivation (eg, “I aspire to know the current sports trend”), and interaction with the environment (eg, “I have strong social skills”). To be deemed physically literate, individuals must exhibit confidence and capability, maintain a positive attitude toward PA [58,59], and effectively interact with their environment through adept self-expression and communication [29]. Responses were gauged on a 5-point Likert scale (1=strongly disagree, 5=strongly agree). Confirmatory factor analysis validated the questionnaire, with factor loadings ranging from 0.60 to 0.92 [60]. All 3 domains demonstrated reliability (Cronbach α=0.87, 0.83, and 0.86).

#### PA and Sedentary Behavior

PA levels were assessed using items from the International Physical Activity Questionnaire-Short Form, which measures the intensity and duration of vigorous PA, MPA, and walking over the past 7 days [61,62]. Metabolic equivalent (MET) task scores were calculated based on the International Physical Activity Questionnaire scoring protocol [63], using a weighted total (vigorous=8.0, moderate=4.0, walking=3.3) to estimate the MET of the task (min/wk). The higher the minutes, the greater the PA level. This instrument assesses 3 levels of PA based on METs, and sedentary behavior (ie, sitting time) was also recorded.

Participants reported the frequency and duration of each PA level, with activities lasting at least 10 minutes being counted. Total weekly PA was calculated using the following formula: *total MET minutes per week* *= walking PA (METs × min × days) + MPA (METs × min × days) + vigorous PA (METs × min × days)* [62]. Sedentary behavior was assessed through a single question about the weekly hours spent sitting. The Chinese questionnaire has been validated in previous studies (age range 15-55 years) and exhibits good test-retest reliability (intraclass correlation coefficient=0.79) [64].

### Statistical Analysis

Data were analyzed using SPSS (version 28; IBM Corp) [65] and the PROCESS macro version 4.0 [66], a tool for mediation and moderation analysis. Two-tailed tests were used, with *P* values <.05 indicating statistical significance. Descriptive statistics described participant demographics, eHEALS, PA and sedentary behavior, and PL scores as means, (SDs) or frequencies (percentages). Two-tailed *t* tests evaluated gender differences in continuous and categorical variables. All continuous variables in the regression model were standardized.

ANOVAs assessed the significance of scores for participants in medicine and sports science. Normality, linearity, and homogeneity of variance were verified before analysis. Following the Pearson correlation, multiple linear regression analyzed the relationships among eHealth literacy, PA, and PL.

Mediation analysis followed established guidelines [67,68], requiring several conditions: (1) a significant path from eHealth literacy to MVPA (path c); (2) a significant association of eHealth literacy with PL (path a); (3) a significant path from PL to MVPA when controlling for eHealth literacy (path b); and (4) a significant indirect effect of PL (a×b) on the relationship between eHealth literacy and MVPA. Bootstrapping with 5000 samples assessed the 95% bias-corrected CI for the indirect effect (Preacher and Hayes [66]). A significant mediating effect was indicated if the CI for the indirect effect did not include zero. Separate regression analyses for gender differences in eHealth literacy PA and PL were also conducted due to observed variations.

## Results

### Participant Characteristics

The number of responses exceeded the anticipated sample size of 381 per university, reflecting high participant interest. In total, 410 responses were distributed to Beijing Normal University and 400 each to Beijing Jiaotong University and Beijing University of Traditional Chinese Medicine, with a total of 1210 questionnaires. Of these, 22 responses were not returned and 64 responses were incomplete, leaving 1124 usable responses (female, n=488, 43.4%; male, n=636, 56.6%). Overall, 86 (7.1%) of the 1210 responses were incomplete and subsequently excluded, leaving 389 responses from Beijing Normal University, 358 responses from Beijing Jiaotong University, and 377 valid responses from Beijing University of Traditional Chinese Medicine. Table 1 illustrates that participant ages ranged from 17 to 28 years, with a mean age of 19.46 (SD 1.58) years. Of the 1124 participants, a majority (n=758, 67.4%) were freshmen, and a smaller group (n=191, 16.9%) were sophomores. Regarding academic disciplines, the majority were science majors (n=726, 64.6%), followed by sports science (n=181, 16.1%), medical studies (n=137, 12.2%), and social sciences (n=80, 7.1%). In this study, “Science” refers specifically to traditional natural sciences, including disciplines such as physics, chemistry, and biology. Geographically, 70.7% (n=795) of participants originated from urban areas, while 29.3% (n=329) came from rural regions.

**Table 1:** Descriptive statistics of participants’ characteristics.

Variables		Value (n=1124), n (%)
**Gender, n (%)**		
	Female	488 (43.4)
	Male	636 (56.6)
**Age (years), mean (SD)**		19.46 (1.58)
**Education level, n (%)**		
	Freshman	758 (67.4)
	Sophomore	191 (16.9)
	Junior	94 (8.4)
	Senior	43 (3.8)
	Postgraduate	38 (3.4)
**Major, n (%)**		
	Social Science	80 (7.1)
	Medicine	137 (12.2)
	Sports Science	181 (16.1)
	Science	726 (64.6)
**Residence, n (%)**		
	Urban area	795 (70.7)
	Rural area	329 (29.3)
**eHealth literacy, mean (SD)**		32.63 (6.40)
**Physical literacy, mean (SD)**		31.68 (6.72)
**Walking MET^a^** **minutes per week, mean (SD)**		922.71 (1204.94)
**Moderate MET minutes per week, mean (SD)**		498.76 (865.55)
**Vigorous MET minutes per week, mean (SD)**		1181.2 (1961.24)
**Moderate to vigorous physical activity MET minutes per week, mean (SD)**		1679.96 (2619.45)
**Total physical activity MET minutes per week, mean (SD)**		2602.67 (3245.53)
**Sitting total minutes per week, mean (SD)**		2398.93 (1239.18)
**Average sitting total minutes per day, mean (SD)**		342.70 (177.03)

^a^MET: metabolic equivalent.

### Descriptive Characteristics of eHealth Literacy and PL

Table 2 presents the average eHealth literacy score as 32.63 (SD 6.40), ranging from 8 to 40. It was noted that participants generally understood the availability of health resources on the internet (mean score, 4.12, SD 0.86), but scored lowest on using eHealth information for decision-making (mean score, 4.05, SD 0.87).

The mean score for total PL was 31.68 (SD 6.72), as detailed in Table 2. This encompassed 3 areas: confidence and physical competence (mean 11.89, SD 2.57), motivation (mean 12.63, SD 2.44), and interaction with the environment (mean 7.89, SD 1.83). Among these, the highest scoring item was awareness of the health benefits of sports (mean 4.32, SD 0.83), while the lowest was physical fitness relative to age (mean 3.73, SD 1.12).

**Table 2:** Descriptive statistics of eHealth literacy and physical literacy for university students.

Item					Value, mean (SD)
**eHealth literacy (range 8-40)**					32.63 (6.40)
	**Awareness**			8.19 (1.67)	
		I know what health resources are available on the internet	4.12 (0.86)		
		I know where to find helpful health resources on the internet	4.07 (0.88)		
	**Skills**			12.23 (2.52)	
		I know how to find helpful health resources on the internet	4.08 (0.88)		
		I know how to use the internet to answer my questions about health	4.08 (0.88)		
		I know how to use the health information I find on the internet to help me	4.07 (0.88)		
	**Evaluation**			12.20 (2.47)	
		I have the skills I need to evaluate the health resources I find on the internet	4.07 (0.86)		
		I can tell high-quality health resources from low-quality health resources on the internet	4.08 (0.85)		
		I feel confident in using information from the internet to make health decisions	4.05 (0.87)		
**Physical literacy (range 8-40)**					31.68 (6.72)
	**Confidence and physical competence**			11.89 (2.57)	
		I possess adequate fundamental movement skills	4.21 (0.88)		
		I am physically fit, in accordance to my age	3.73 (1.12)		
		I am able to apply learned motor skills to other physical activities	3.95 (0.94)		
	**Motivation**			12.63 (2.44)	
		I appreciate myself or others doing sports	4.23 (0.89)		
		I am aware of the benefits of sports related to health	4.32 (0.83)		
		I aspire to know the current sports trend	4.07 (1.00)		
	**Interaction with the environment**			7.89 (1.83)	
		I have strong communication skills	3.96 (0.98)		
		I have strong social skills	3.94 (1.03)		

### Different Majors in PL, PA, and eHealth Literacy

#### Descriptive Statistics of the PA-Related Variables, PL, and eHealth Literacy

Significant differences in walking MET, MPA MET, vigorous PA MET, MVPA MET, and PL scores were observed across different majors, as shown in Table 3, although no significant variation in eHealth literacy scores was detected (*P*=.14).

**Table 3:** Descriptive statistics of physical activity, physical literacy, and eHealth literacy in different academic majors.

Item	Major, mean (SD)					*F* test (*df*)		*P* value
	Social Science (n=80)	Science (n=726)	Medicine (n=137)	Sports Science (n=181)				
Total MVPA^a^	1683.3 (2370.4)	1471.5 (2508.5)	823.1 (1161.0)	3163.3 (3321.6)	27.6 (3, 1120)		<.001	
VPA^b^-MET^c^ minutes per week	1136.8 (1626.5)	1016.7 (1886.2)	553.3 (868.9)	2336.1 (2487.1)	29.4 (3, 1120)		<.001	
MPA^d^-MET minutes per week	546.5 (902.6)	454.8 (849.2)	269.8 (421.3)	827.2 (1058.5)	13.0 (3, 1120)		<.001	
Walking-MET minutes per week	837.0 (1046.4)	924.4 (1236.0)	729.0 (1053.8)	1100.4 (1233.7)	2.6 (3, 1120)		.048	
Physical literacy	31.7 (7.0)	31.4 (6.7)	30.2 (6.7)	33.8 (6.3)	8.7 (3, 1120)		<.001	
eHealth literacy	32.3 (6.4)	32.6 (6.5)	31.9 (5.9)	33.5 (6.5)	1.9 (3, 1120)		.14	

^a^MVPA: moderate to vigorous physical activity.

^b^VPA: vigorous physical activity.

^c^MET: metabolic equivalent.

^d^MPA: moderate physical activity.

#### Distribution of PA Levels Across Different Majors

Different majors in PA levels are shown in Table 3, which indicates that sports science majors reported the highest weekly MVPA (3163.3 MET min), in contrast to medicine majors who reported the lowest (823.1 MET min). Regarding low-intensity PA, like walking, medical students recorded the lowest levels (729 MET min), followed by those in social sciences (837 MET min), science (924.42 MET min), and sports science (1010.4 MET min).

#### Analysis of eHealth Literacy and PL Among Students Majoring in Sports Science and Medicine

Table S1 in Multimedia Appendix 1 shows significant differences in eHEALS (*P=*.02) and PL (*P*<.001) among sports science students and medicine major students. It also shows significant differences in confidence and physical competence (*P<*.001), interaction with the environment (*P<*.001), total PL (*P<*.001), and motivation (*P=*.002), in sports science and medicine students.

### Gender Difference in PL, PA, and eHealth Literacy

#### The Distribution of PA Levels Across Different Genders

PA levels were categorized into 3 groups: low, moderate, and high. Low PA was defined as less than 600 MET minutes per week, high PA as more than 3000 MET minutes per week, and MPA as between 600 and 3000 MET minutes per week. Table S2 in Multimedia Appendix 1 indicates that nearly half (554/1124, 49.3%) of the participants fell into the MPA category. Among them, 18.1% (115/636) of male students were at the low level, while a higher proportion of female students (129/488, 26.4%) were classified as having low PA. The percentage of individuals at the high PA level was greater for male students (224/636, 35.2%) compared to female students.

#### Analysis of PA, PL, and eHealth Literacy in Different Genders

Table S3 in Multimedia Appendix 1 presents the differences between male and female students in BMI, PA, PL, and eHEALS. The vigorous physical activity, MPA, MVPA, and walking MET were significantly different between these 2 groups (*P*<.001). However, there were no significant differences for eHealth literacy (*P*=.23), and male participants had a higher BMI and PL than female participants.

#### Correlations Between PL, MVPA, and eHealth Literacy in Different Genders

Pearson correlations between PL, MVPA, and eHealth literacy are presented in Table S4 in Multimedia Appendix 1. eHealth literacy and MVPA were significantly correlated with PL (*P*<.001), and eHealth literacy was also significantly associated with MVPA (*P*<.001).

### Mediation Model Test

Multiple linear regression was used to evaluate the relationships between eHealth literacy, PL, and MVPA, as outlined in Table 4. All identified correlations were positive and robust, confirming significant associations among these variables. The regression models formulated were as follows: *PL = 5.57 + 0.80 × eHealth Literacy*, *MVPA = –390.64 + 63.46 × eHealth Literacy*, and *PL = 30.711 + 0.001 × MVPA*.

**Figure 1:**
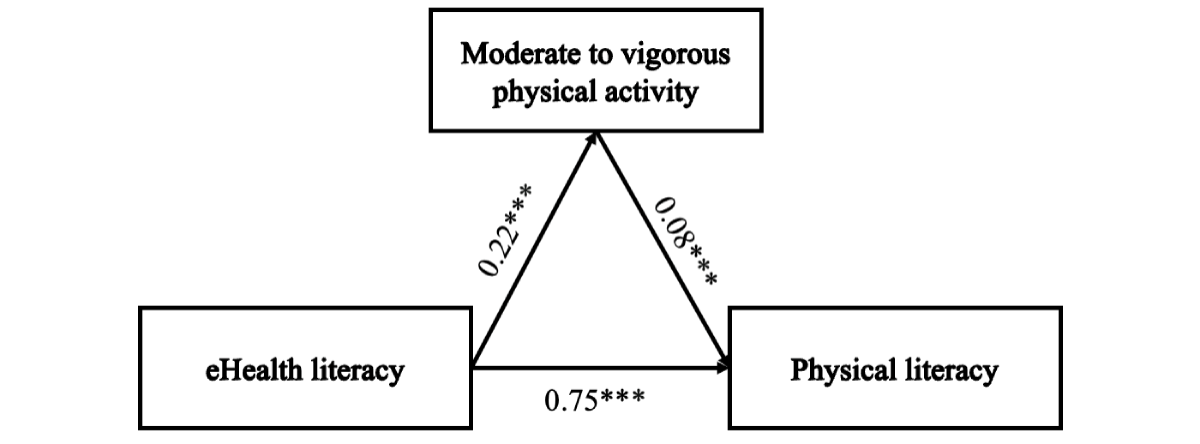
Model 1. Relationship between eHealth literacy and physical literacy with moderate to vigorous physical activity as a mediator (***P<.001).

In model 1, significant pathways were observed from eHealth literacy to PL (β .75, SE 0.02; *P*<.001) and from eHealth literacy to MVPA (β=.22; *P*<.001), as depicted in Figure 1. Additionally, a significant link was found from MVPA to PL (β=.08; *P*<.001; Figure 1). Table 5 displays the mediating role of PA in the interaction between PL and eHealth literacy. The mediation analysis indicated a direct effect of eHealth literacy on PL at 0.78 and an indirect effect at 0.18. Furthermore, the total effect was calculated at 0.80. Bootstrap analysis with 5000 samples demonstrated an SE of 0.01 and a 95% bias-corrected CI ranging from 0.01 to 0.29, excluding zero. Thus, MVPA served as a partial mediator, accounting for 2.22% of the total effect in the relationship between eHealth literacy and PL.

**Figure 2:**
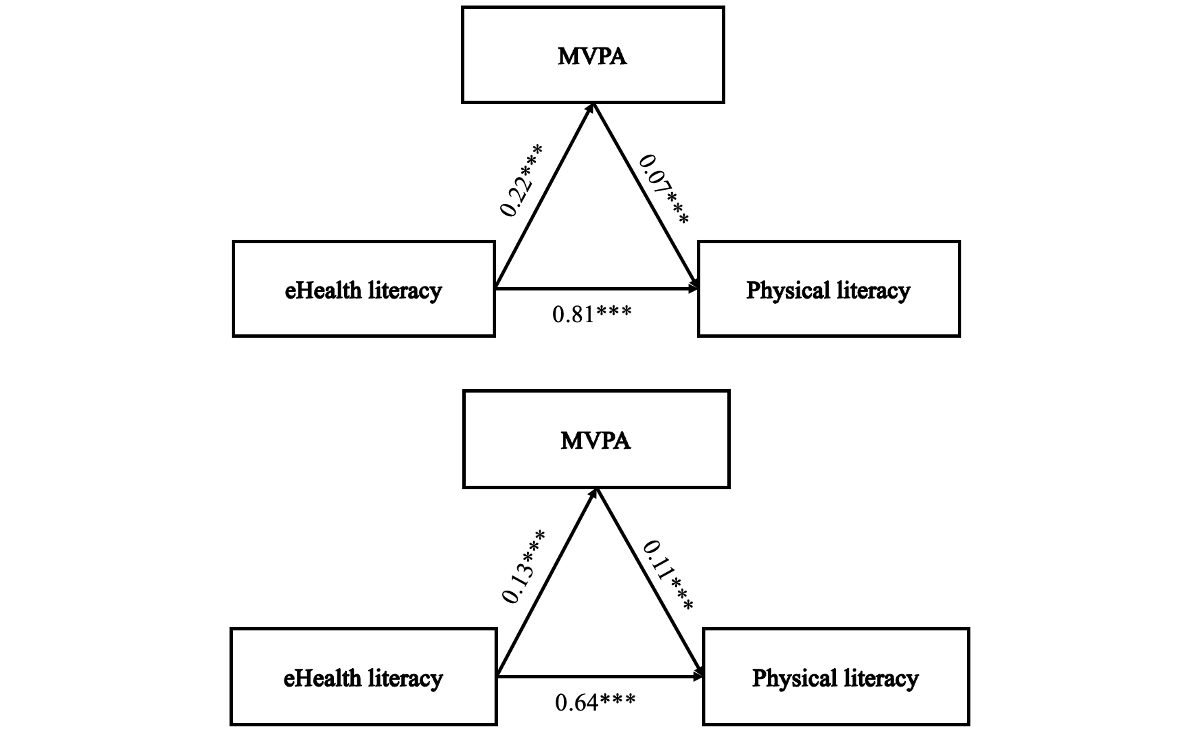
Model 2. The gender difference (male and female) in the relationship between eHealth literacy, physical literacy, and MVPA (***P<.001). MVPA: moderate to vigorous physical activity.

Model 2 explored the mediating effect of MVPA on the relationship between eHealth literacy and PL across genders, as visually represented in Figure 2 For male students, significant paths from eHealth literacy to PL (β=.81; *P<*.001) and from eHealth literacy to MVPA (β=.22; *P<*.001) were noted. For female students, eHealth literacy was significantly associated with both PL (β=.64; *P<*.001) and MVPA (β=.13; *P*<.001).

Table 5 further delineates the mediation effects within the relationship among eHealth literacy, MVPA, and PL-related variables. The mediation model indicated direct effects of 0.82 for male students and indirect effects of eHealth literacy on PL at 0.02, which were statistically significant. MVPA’s mediating role accounted for 1.84% of the total effect, affirming its position as a partial mediator between eHealth literacy and PL. In female students, the mediation model of eHealth literacy on PL revealed direct effects of 0.717 and indirect effects of 0.016, with MVPA contributing to 2.16% of the total effect.

**Table 4:** Results of the standard linear regression analysis among eHealth literacy, PL^a^, and MVPA^b^.

Participants and item					β (SE)		*F* test (*df*)			95% CI			*R*			Δ*R*^2^
**All**																
	**eHEALS^c^**															
		PL	.75 (0.02)^d^			1558.64 (1, 1122)		0.76-0.84			0.77			0.59		
		MVPA	.22 (12.06)^d^			27.68 (1, 1122)		39.79-87.13			0.76			0.58		
	**MVPA**															
		PL	.08 (0.00)^d^			59.63 (1, 1122)		0.0004-0.0007			0.21			0.05		
**Male students**																
	**eHEALS**															
		PL	.81 (0.02)^d^			1333.42 (1, 634)		0.79-0.88			0.82			0.68		
		MVPA	.22 (16.71)^d^			17.05 (1, 634)		36.18-101.80			0.16			0.03		
	**MVPA**															
		PL	.07 (0.0001)^d^			33.64 (1, 634)		0.0004-0.0007			0.22			0.05		
**Female students**																
	**eHEALS**															
		PL	.64 (0.04)^d^			370.08 (1, 486)		0.66-0.81			0.66			0.45		
		MVPA	.13 (15.89)^d^			8.13 (1, 486)		14.10-76.56			0.13			0.17		
	**MVPA**															
		PL	.11 (0.0002)^d^			18.80 (1, 486)		0.0001-0.0006			0.19			0.04		

^a^PL: physical literacy.

^b^MVPA: moderate to vigorous physical activity.

^c^eHEALS: eHealth literacy scale.

^d^Correlation is significant at the .01 level (2-tailed).

**Table 5:** Mediating effects of MVPA^a^ on the relationship among eHealth literacy and PL^b^ variables.

Mediating effect of MVPA on model	Direct effect				Indirect effect				Mediating effect (%)		
	All	Male	Female	All		Male	Female	All		Male	Female
eHEALS^c^ and PL	0.78	0.82	0.72	0.18		0.02	0.016	2.22		1.84	2.16
eHEALS and CPC^d^	0.29	0.30	0.27	0.007		0.006	0.007	2.41		2.03	2.35
eHEALS and M^e^	0.26	0.28	0.23	0.004		0.003	0.005	1.36		0.95	2.01
eHEALS and IE^f^	0.20	0.21	0.17	0.003		0.003	0.003	1.65		1.3	1.55

^a^MVPA: moderate to vigorous physical activity.

^b^PL: physical literacy.

^c^eHEALS: eHealth literacy scale.

^d^CPC: confidence and physical competence.

^e^M: motivation.

^f^IE: interaction with the environment.

## Discussion

### Principal Findings

This investigation found robust positive associations between eHealth literacy and PL, supporting hypothesis 1. Additionally, the mediating role of MVPA was instrumental in delineating the connection between eHealth literacy and PL. Male students also showed higher PA and PL than female students, although no significant differences in eHealth literacy were observed. This study is pioneering in examining the linkage between eHealth literacy and PL among Chinese university students and is among the extensive investigations exploring MVPA’s mediating effects. Overall, the findings indicate that both eHealth literacy and MVPA are [69] positively associated with [27,28,70] PL among university students.

### Individuals With Higher eHealth Literacy Tend to Have Greater PL

This study identified eHealth literacy as a significant predictor of PL. Thus, university students with higher eHealth literacy are generally more physically literate. One mechanism linking eHealth literacy to improved PL is PA. Public health initiatives have increasingly focused on enhancing university students’ eHealth literacy and PA, with numerous studies demonstrating that eHealth literacy is positively correlated with health behaviors including regular exercise and PA [27,28,70]. Additionally, both MVPA and health behaviors are strongly associated with PL [38]. The results of this study support the hypothesis that MVPA serves as a partial mediator in the relationship between eHealth literacy and PL. In essence, improving eHealth literacy to increase MVPA may enhance PL among university students.

### eHealth Literacy Prevalence Among Chinese University Students

The average eHEALS score among the students was 32.63, surpassing scores reported in global studies [71-73]. This elevated level of eHealth literacy could be attributed to the enhanced availability of reputable health information sources in China. The Chinese government’s initiatives to disseminate health information via social media and the accessibility of international health websites in Chinese may contribute to this trend [74]. Despite these high scores, many students demonstrated a lack of proficiency in distinguishing between high-quality and low-quality health information sources, often hesitating to make health decisions based on web-based information. These findings align with previous research [75,76].

### Gender and Academic Major Differences in eHealth Literacy

Although male students achieved higher eHEALS scores than female students, the differences were not statistically significant, diverging from some prior studies in Taiwan [21,23,77]. Furthermore, the influence of academic majors on eHealth literacy levels was evident, with Chinese Medicine students displaying the lowest eHealth literacy. This finding contrasts with prior research indicating higher eHealth literacy among Western medical students [18,24]. The intensive curriculum and numerous internships in traditional Chinese medicine programs, which involve extensive memorization of classical texts, may reduce these students’ engagement with digital health resources. However, sports science students recorded higher eHEALS scores (33.5), a significant variance, supporting social cognitive theory [78] that environmental factors can influence health perceptions [79].

### PA and PL Among Male Students

Consistent with previous research, this study found that PL influences PA levels, and participation in PA substantially affects PL [33]. An intriguing aspect of this study was the higher motivation for PA among university students, who exhibited lower confidence and competence in their physical activities. Moreover, gender differences in PL and PA were noted, with male students displaying higher levels than female students, corroborating findings from a study in southern China on the interplay between PL and PA [60]. Globally, men tend to be more physically active than women, with physical inactivity being more prevalent among women in nearly all countries [1,3]. Consequently, it is crucial to establish PA habits and motivate university students, particularly female students, to develop a lifelong commitment to PA.

A significant disparity was observed in PL and PA between medical students and sports science students. These findings align with a large quantitative study conducted in German universities assessing PA levels [69]. Sports science students, being more aware of the potential health consequences of physical inactivity, are more inclined to be physically active. Therefore, it is recommended that medical students increase their PA participation and engage in PL-related interventions and curricula. Further research should evaluate eHealth literacy in a larger cohort of Chinese university students majoring in medicine or health sciences.

### Implications

Our findings have significant implications for educational practices in universities. Health educators are urged to enhance health education and develop robust health education programs to enhance overall health outcomes for university students. Given that physical education is mandatory in mainland China from primary school to university levels [80], integrating eHealth literacy into physical education classes could support holistic student development in PL. Additionally, incorporating content on MVPA into curricula could support students’ PA and literacy levels. It is imperative for university-level eHealth interventions to use mobile health apps social media, and various digital resources to enhance students’ skills in navigating and evaluating web-based health information [8].

Furthermore, scholars should investigate the factors contributing to gender disparities in PA and PL. Targeted health education programs at universities could significantly benefit female students by enhancing their PA and PL. Additionally, students in health-related fields, particularly medical students, require targeted educational interventions to ensure comprehensive mastery of eHealth resources. Previous studies indicate that women are more physically inactive [81,82] and have lower PL [38]. Therefore, enhancing their eHealth literacy and promoting PA could significantly improve their health outcomes.

### Strengths and Limitations

This study is distinguished by its diverse participant base and the use of advanced analytical methods to elucidate MVPA’s role in the dynamics between eHealth literacy and PL. However, it also has limitations. The participant pool was confined to 3 universities, suggesting that future research could broaden the scope to include more institutions. Moreover, the reliance on self-reported data for PA, PL, and eHealth literacy could introduce biases; subsequent studies might benefit from more objective data collection methods. Additionally, the cross-sectional design precludes causal inferences between eHealth literacy, MVPA, and PL. Finally, data collection coincided with the winter holiday, potentially affecting the typical PA levels of students.

### Conclusions

This study elucidated the relationships among eHealth literacy, PL, and PA in a cohort of Chinese university students, highlighting the mediating role of MVPA. The results suggested that students with higher eHealth literacy and PA levels are likely to exhibit enhanced PL. Sports science students outperformed medical students in eHealth literacy, PL, and PA levels. Moreover, male students were more active and physically literate than female students, yet no significant gender differences in eHealth literacy were detected. Future research should aim to validate these findings through longitudinal studies and consider eHealth literacy and MVPA when designing PL intervention programs for university students.

## Appendix

Multimedia Appendix 1 Supplementary tables.

## Abbreviations

eHEALS: eHealth Literacy Scale
MET: metabolic equivalent
MPA: moderate physical activity
MVPA: moderate to vigorous physical activity
PA: physical activity
PL: physical literacy
WHO: World Health Organization

## Data Availability

The datasets generated during and analyzed during this study are available from the corresponding author upon reasonable request.
