# Supplementary material for: Relationships Among eHealth Literacy, Physical Literacy, and Physical Activity in Chinese University Students: Cross-Sectional Study
Source: J Med Internet Res. 2024 Nov 4;26:e56386. doi: 10.2196/56386 (PMC11574492; doi:10.2196/56386)
Supplement: Multimedia Appendix 1 [file jmir_v26i1e56386_app1.docx]

**Table S1.** Analyses of physical literacy and eHealth literacy scores among students majoring in medicine and sports science

| Item |  | Major, mean (SD) | | *t* test (*df*) | *P* value^a^ |
| --- | --- | --- | --- | --- | --- |
|  |  | Medicine (n=137) | Sports Science (n=181) |  |  |
| eHealth literacy |  | 31.9 (5.9) | 33.5 (6.5) | -2.3 (316) | .021 |
|  | Awareness | 8.0 (1.6) | 8.4 (1.7) | -2.2 (316) | .029 |
|  | Skills | 12.0 (2.4) | 12.5 (2.6) | -1.7 (316) | .085 |
|  | Evaluation | 11.9 (2.2) | 12.6 (2.5) | -2.8 (316) | .006 |
| Physical literacy |  | 30.2 (6.7) | 33.8 (6.3) | -4.9 (316) | <.001 |
|  | Confidence and physical competence | 11.4 (2.6) | 12.7 (2.4) | -4.8 (316) | <.001 |
|  | Motivation | 12.3 (2.6) | 13.1 (2.4) | -3.0 (316) | .002 |
|  | Interaction with the environment | 7.5 (1.9) | 8.4 (1.6) | -4.6 (316) | <.001 |

^a^*P* value: two-sided *P*.

**Table S2.** Gender difference between eHealth literacy, PL^a^, and PA^b^.

| PA level | Male (n=636) | Female (n=488) | Total (n=1124) |
| --- | --- | --- | --- |
| Low, n (%) | 115 (18.1) | 129 (26.4) | 244 (21.7) |
| Moderate, n (%) | 297 (46.7) | 257 (52.7) | 554 (49.3) |
| High, n (%) | 224 (35.2) | 102 (20.9) | 326 (29.0) |

^a^PL: physical literacy.

^b^PA: physical activity.

**Table S3.** Gender difference between eHealth literacy, PL^a^, and PA^b^.

| Item | Male (n=636), mean (SD) | Female (n=488), mean (SD) | *t* test (*df*) | *P* value |
| --- | --- | --- | --- | --- |
| BMI | 22.42 (3.41) | 20.83 (2.98) | 8.36 (1122) | <.001 |
| Walking-MET^c^ minutes per week | 1012.22 (1313.41) | 806.05 (1036.63) | 2.94 (1122) | .003 |
| MPA^d^-MET minutes per week | 604.44 (975.89) | 361.02 (672.46) | 4.94 (1122) | <.001 |
| VPA^e^-MET minutes per week | 1465.35 (2184.93) | 810.89 (1549.89) | 5.62 (1122) | <.001 |
| MVPA^f^ MET minutes per week | 2069.79 (2958.65) | 1171.91 (1987.81) | 6.07 (1122) | *<*.001 |
| Physical literacy | 32.34 (7.00) | 30.95 (6.27) | 3.18 (1122) | .002 |
| eHealth literacy | 32.83 (6.94) | 32.37 (5.63) | 1.19 (1122) | .23 |

^a^PL: Physical literacy.

^b^PA: physical activity.

^c^MET: metabolic equivalent task.

^d^MPA: moderate physical activity.

^e^VPA: vigorous physical activity.

^f^MVPA: Moderate to vigorous physical activity.

**Table S4**. Pearson correlations between PL, MVPA^a^, and eHealth literacy

| Variable | Gender | 1 | 2 | 3 | 4 | 5 | 6 | 7 | 8 | 9 | 10 |
| --- | --- | --- | --- | --- | --- | --- | --- | --- | --- | --- | --- |
| (1)BMI | Male | ­­­— |  |  |  |  |  |  |  |  |  |
|  | Female | — |  |  |  |  |  |  |  |  |  |
| (2)MVPA | Male | 0.05 | — |  |  |  |  |  |  |  |  |
|  | Female | -0.08 | — |  |  |  |  |  |  |  |  |
| (3)Confidence and physical competence | Male | 0.03** | .23** | — |  |  |  |  |  |  |  |
|  | Female | -0.13 | .20** | — |  |  |  |  |  |  |  |
| (4)Motivation | Male | 0.02 | .17** | .89** | — |  |  |  |  |  |  |
|  | Female | -0.05 | .16** | .76** | — |  |  |  |  |  |  |
| (5)Interaction with the environment | Male | 0.05 | .19** | .87** | .82** | — |  |  |  |  |  |
|  | Female | -0.05 | .14** | .73** | .67** | — |  |  |  |  |  |
| (6)Total physical literacy | Male | 0.03 | .22** | .99** | .89** | .93** | — |  |  |  |  |
|  | Female | -.11* | .19** | .98** | .78** | .85** | — |  |  |  |  |
| (7)Awareness | Male | .09* | .15** | .79** | .76** | .77** | .81** | — |  |  |  |
|  | Female | -0.05 | .14** | .60** | .55** | .51** | .61** | — |  |  |  |
| (8)Skills | Male | .11** | .16** | .78** | .75** | .76** | .80** | .96** | — |  |  |
|  | Female | -0.06 | .14** | .59** | .52** | .52** | .60** | .91** | — |  |  |
| (9)Evaluate | Male | .09* | .17** | .79** | .73** | .78** | .81** | .91** | .91** | — |  |
|  | Female | -0.04 | 0.08 | .61** | .53** | .57** | .64** | .78** | .78** | — |  |
| (10)eHealth literacy | Male | .10* | .16** | .81** | .76** | .79** | .82** | .98** | .98** | .97** | — |
|  | Female | -0.05 | .13** | .64** | .57** | .57** | .66** | .94** | .96** | .92** | — |

* *P*<.05, ** *P*<.001 (two-tailed)

^a^MVPA: Moderate to vigorous physical activity.
